# Identifying Risk Factors for Pulmonary Tuberculosis Diagnosis Delays in Mali a West-African Endemic Country

**DOI:** 10.4236/jtr.2022.101004

**Published:** 2022-03-25

**Authors:** Dianguina Soumare, Bocar Baya, Khadidia Ouattara, Tenin Kanoute, Cheick M. Sy, Seydou Karembé, Ibrahima Guindo, Lamine Coulibaly, Youssouf Kamian, Aime P. Dakouo, Fatoumata Sidibe, Salif Koné, Drissa Kone, Oumar Yossi, Gaoussou Berthe, Yacouba Toloba

**Affiliations:** 1 Service of Pneumology of the University Teaching Hospital of Point-G, Bamako, Mali; 2 University Clinical Research Center (UCRC), University of Sciences, Techniques and Technologies of Bamako (USTTB), Bamako, Mali; 3 Non-Government Organization: Sante-Sud, Bamako, Mali.

**Keywords:** Tuberculosis, Diagnosis, Delays, Risk-Factors, Mali

## Abstract

**Background:**

Tuberculosis was the deadliest infectious agent before covid-19; 1.5 million deaths in 2020. Despite, a variety, of easy and cheap diagnostic tools, detection rates still fall below 90%; diagnosis delays are long exceeding 30 days in many continents. This study aimed to determine risk factors for pulmonary TB diagnosis delays in Mali.

**Methods:**

A cross-sectional study was conducted in Bamako to include pulmonary TB patients at treatment initiation centers. Verbal consent was obtained before the interview. Demographics, clinical, treatment cost, and patient, medical, and diagnostic delays were computed using SPSS 25.0 considering a significance level p < 0.05.

**Results:**

In total 266 patients were included, 80.8% were male, mean age was ± 12 years, primary education level was 50.4%, treatment cost before diagnosis was 100 - 200 thousand CFA in 65.4%, smokers were 42.1%, median patient, medical and total diagnostic delays were 58, 57 and 114 days respectively. Education level below university, social reasons, and non-request of health workers were identified as independent risk factors for diagnostic delay > 100 days in Mali

**Conclusion:**

Diagnostic delay is relatively very long in Mali, there is an urgent need for identification and action to shorten the delays to limit the transmission chain and avoid disabling pulmonary sequels.

## 1. Introduction

Tuberculosis (TB) is a community-acquired disease whose infectious agent was before the COVID-19 pandemic the leading cause of death from a single infectious agent worldwide [1]. Detection rates are still under 90% targeted by World Health Organization (WHO) for TB elimination [2]. In 2020, the number of newly diagnosed cases was 5.8 million, far from the 10 million estimated cases worldwide meaning a decline of 18% attributable to the effect of the pandemic of COVID-19 [1]. Longer diagnosis delays contribute to the spread of TB infection. Several strategies for TB detection and treatment have been implemented in countries for several years such as the Directed Observed Treatment Strategy (DOTS), still, many TB cases continue to escape the health system, leading either to irreversible complications or death. In 2020, 1.5 million people died of tuberculosis, including 214,000 cases of TB/HIV co-infection [1]. Lungs are the preferred organ for TB bacteria localization and spread, the delay in diagnosis is a factor of poor prognosis. In the Democratic Republic of Congo (DRC), a study showed that the risk of death was 5 times higher in smear-positive pulmonary tuberculosis than in other forms of tuberculosis [3]. In West Africa, a study conducted in Guinea Conakry on TB diagnosis delay reported a median delay of 11 weeks and a half (54%) of the patients had used non-conventional medicine [4]. A systematic review and meta-analysis reported that in sub-Saharan Africa the time from symptom onset to the diagnosis of TB varied between countries, ranging from 4 days in Sudan to 63 days in Ethiopia with a median of 28 days [5]. In Kigali, a study found a total diagnosis delay of 57 days and identified that the trial of antibiotics by the healthcare system increases the risk by 3 folds of delaying TB diagnosis [6]. A literature review on common TB diagnosis delay risk factors has reported several modifiable gaps such as low knowledge of TB symptoms, old age, social belief, repeated visits in health centers having the same level, and initial consultation of traditional therapy. In developed countries, the diagnosis of TB is also delayed; in the United Kingdom, a delay of 49 days was reported in London [7]. In Portugal, a median total diagnosis delay of 62 days was observed, and there was an increase in the total diagnosis duration between 2008 and 2017 [8]. In countries with a high burden of TB, longer delays can significantly contribute to the spread of the disease in the community. In India, a total diagnosis delay of 130 days was reported; smoking and being part of the low-income group were found to be risk factors for delayed diagnosis [9]. In Mali, there is a lack of data neither on TB diagnosis delays nor on risk factors and reasons for longer delays, thus, this study aimed to identify risk factors for pulmonary TB diagnosis delays in Mali.

## 2. Methods

### Study Design and Setting.

A cross-sectional analytic study was conducted from January 2019 to December 2020 at the service of pneumology of the University Hospital of Point-G in Bamako, Mali.

### Study Population:

Patients diagnosed with positive microscopy pulmonary TB and treatment initiated at one of the six referral health centers (RHC) of Bamako.

### Inclusion criteria:

Patients who were aged 18 years or more, have been diagnosed with pulmonary TB by sputum microscopy and/or GeneXpert MTB/RIF®, and have verbally consented to participate in the study were interviewed.

### Exclusion criteria:

Patients with a diagnosis of tuberculosis who were not able to participate because of the level of illness and those who did not consent to participate.

### Procedures and data collection:

Patients with sputum positive for Acid-Fast Bacilli (AFB) by microscopy and/or the presence of the genome of *Mycobacterium tuberculosis* (MTB) by GeneXpert MTB/RIF^®^ are routinely referred for anti-tuberculosis treatment initiation. These patients were approached for a brief description of the study, verbal consent, and data collection. Information was collected during a one-on-one interview with the participant using a pre-established questionnaire (appendix). Data collected included socio-demographic variables including age, gender, occupation, marital status, level of education; diseases symptoms; smoking status, chest x-ray results; facilities visited, and the average cost of the expenses before the test that confirmed TB diagnosis.

### Definitions:

Patient delay was defined as the duration between the onset of TB symptoms (cough plus one of the following symptoms night fevers and/or sweat, weight loss) and the first medical consultation (healthcare visit). The medical (health) delay, the duration between the first consultation at a medical center and the TB diagnosis. The diagnostic (total) delay, the period between symptoms onset, and the diagnostic test result. The diagnosis delay was the sum of patient delay and medical delay.

### Data analysis:

Data were de-identified, entered in Excel 2010, cleaned, and analyzed with SPSS 25.0 for Windows. After sample description of sample characteristics, ANOVA test was performed to compare means, Chi^2^ test was used to determine associations, a logistic regression analysis was performed including variables that had an association with diagnosis (total) delay of more than 100 days with a p-value ≤ 0.25. Any difference was seen with a probability of p < 0.05 was considered statistically significant.

### Ethical Considerations:

An agreement to conduct the study was provided by the Director of the University Teaching Hospital of Point G before the study was conducted. A Verbal consent was obtained from each patient before data collection. Data were anonymized before analysis and results are presented in aggregates format.

## 3. Results

A total of 266 patients were included in this study from January 2019 to December 2020. Males were predominant with 80.8%. The age group [26 - 50] years represented 75.2% with a mean age of 40.5 ± 11.9 years, half (50.4%) of the patients had a primary level of education, 74.1% were residents in Bamako and 25.9% came from different regions of Mali. Two-thirds of patients (65.4%) had a monthly income between 100,000 and 200,000 CFA around 180 - 360 US Dollars. Smoking was found in 42.1% of whom 81.2% had a tobacco consumption between 0 and 20 packs/year. Clinical symptoms found in addition to cough were dyspnea (63.5%), chest pain (76.3%), and hemoptysis (12.8%). The body mass index (BMI) was below 18.5 kg/m^2^ in 79.7%. The WHO performance status (PS) score was either 1 or 2 in 66.2%. Chest X-ray showed bilateral infiltrates in 50.4% and excavation was present in 17.7% (Table 1). Consultation of traditional health providers or self-medication was found in 44.4%. Before TB diagnosis 53.0% consulted in 1 to 2 health facilities and 47.0% in 3 or more clinics. Among health structures visited, 60.9% were communal health district (CSCOM), pharmacy or dispensary (44.0%), and private clinics (39.9%). Different reasons were provided by the patients to justify the time between symptoms onset and the diagnosis, test non requested by a health worker (medical reason) was the most found (79.7%), personal reasons (38.3%), and financial reasons (33.5%), social reasons (30.8%) and professional reasons (30.5%). The estimated cost of patients’ expenses before diagnosis was between 50,000 and 100,000 CFA in 35.7% and spent more than 100,000 CFA (180 US Dollars) in 47.7%. The patient delay was between 31 - 60 days in 61.3% with an average of 58.1 ± 19.4 days (median = 58.2 days); the medical (health) delay was between 31 - 60 days in 62.8% with a mean of 57.2 ± 19.9 days (median = 57.1 days); the diagnostic (total) delay was between 120 - 150 days (4 - 5 months) in 69.6% with a mean of 115.0 ± 23.5 days (median = 114.2 days) ~4 months (Table 2). Comparing means diagnosis delay and cost of expenses before diagnosis. There was a statistically significant difference between the mean of the diagnosis delay and the cost. Those who spent less than 50,000 CFA had a mean delay of 44.4 ± 19.9 days; patients who spent 50,000 - 100,000 CFA had a mean delay of 56.53 ± 16.72 and those who spent more than 100,000 CFA had a mean delay of 61.5 ± (p < 0. 00001). Among the 112 smokers, 50.0% (65/112) had spent more than 100,000 FCFA before TB diagnosis; a statistically significant association was observed between cost and smoking, p = 0.009 (Table 3). Comparing mean diagnosis delay between patients who seek or not for traditional medicine consultation, there was a statistically significant difference between the mean patient delays, there were more days for patients who visited traditional therapy 6.84 ± 2.35 95% CI (2.19 - 11.48), p = 0.004. This difference affected the diagnosis delay with a difference of 10.04 ± 2.83 95% CI (4.43 - 15.65) days, p < 0.000001. Univariate analysis was performed (Table 4), primary healthcare/pharmacy [OR = 0.35 (0.20 - 0.60), p < 0.0001], traditional therapy or Self-medication [OR = 2.24 (1.30 - 3.83), p = 0.004], Hemoptysis [OR = 16.07 (2.16 - 119.78), p < 0.0001], Dyspnea [OR = 6.66 (3.72 - 11.92), p < 0.00001]. Financial reason [OR = 2.17 (1.18 - 4.01), p = 0.015], Social reason [2.83 (1.46 - 5.51), p = 0.002] and Health worker did not request for TB test [2.17 (1.17 - 4.04), p = 0.018] were factors associated with a diagnosis delay longer than 100 days. Logistic regression was performed to determine risk factors. Education level below university [aOR = 9.7 (1.9 - 50.2), p = 0.007]; social reasons [aOR = 3.4 (1.2 - 9.4), p = 0.021] and the non-request of TB test by health workers [aOR = 8.1 (2.8 - 22.9), p < 0.0001] were independent risk factors associated with long delay of more than 100 days before diagnosis of tuberculosis (Table 5).

## 4. Discussion

### 4.1. Diagnosis Delays among Tuberculosis Patients in Health Practices

Tuberculosis is one of the deadliest curable diseases [1]. Its late diagnosis can lead to death or pulmonary complications. The duration between symptoms on-set and diagnosis is crucial for breaking the transmission chain. A longer duration increases the risk of disease transmission, death, and irreversible respiratory complications such as chronic respiratory failure. This study found a predominance of males (80.8%), two-thirds were aged between 26 - 50 years with a mean of 40.5 ± 11.9 years, half had a level of primary education, more than half had a monthly income between 100 and 200 thousand CFA francs (~180 - 360 $US). Smoking was found in 42.1%, dyspnea 63.5%, lung infiltrates were bilateral in half of the cases (50.4%) with excavation in 17.7%. More than one-third (44.4%) had self-medication and or consulted a traditional doctor, half (53.0%) of patients visited 1 or 2 health facilities. The median patient delay (58.2 days) was comparable to the median health delay (57.1 days) while the median diagnosis delay was longer (114.2 days - 4 months). The median delays found in this study are relatively very long compared to those of several studies in the world. Thus, in Africa, a Tanzanian study reported a median diagnostic delay of 3 weeks [10]. In Ethiopia, three studies reported respectively a median patient delay (30 days), healthcare system delay (21 days) in 2005 [11]; median patient delay (20 days), health delay (33.5 days), and a total diagnosis delay of 70.5 days [12] in 2012 and lastly in 2020, the median of patient delay was 35 days and 54.4% of patients had their first consultation after 21 days [13]. In Gambian, a median duration of 34 days was found from symptoms onset to diagnosis [14]. A Central African reported in a study with 58.2% of females (mean age 35.7 ± years), a mean diagnosis delay of 37.7 days [15]. In South Africa, a study reported among HIV-infected patients, a diagnostic delay of 31 - 180 days in 33.8%, and in 31.2% the delay exceeded 180 days [16]. None of these studies has reported a diagnostic delay below a month inferring that TB diagnostic is made relatively late in Africa. In other continents, the overall diagnostic delays are not different from those in the African continent. In Nepal, the median patient delay was 50 days, the median health system-related delay was 18 days, and the median total diagnosis delay was 60 days [17]. In Hong Kong, the risk of mortality is associated with delayed diagnosis in elderly patients, 33.7% of TB-related deaths occurred at the time of diagnosis [18]. However, this study did not consider mortality from tuberculosis. In China, the median diagnosis of TB was significantly longer among immigrants than natives (30 vs. 9) with a high proportion of patients with a duration of >28 days (52% vs. 13%) [19]. In another Chinese study, the time from the first consultation to diagnosis was more than 30 days in 25% of kidney transplanted patients [20]. In Cambodia, a median diagnosis delay of 49 days was found in TB patients [21]. Again, in the Asian continent, the diagnosis delay was comparable to that of Africa. In the United States, a study conducted by the Harvard Medical School researchers reported a mean diagnosis delay of 24 days [22]. This can infer that even in extremely developed and well-equipped countries TB diagnoses are delayed.

### 4.2. Reasons and Risk Factors Associated with a Longer Delay in Tuberculosis Diagnosis

In our study, several reasons were provided by patients as having contributed to the diagnosis delay, including medical reasons (79.7%), personal (38.3%), financial (33.5%), social (30.8%), and professional (30.5%). Education level below university [aOR = 9.7 (1.9 - 50.2), p = 0.007]; social reasons [aOR = 3.4 (1.2 - 9.4), p = 0.021] and test was not request by the health worker [aOR = 8.1 (2.8 - 22.9), p < 0.0001] were identified as independent risk factors for delayed diagnosis of tuberculosis above 100 days in Mali. In African studies, in The Gambia, age between 18 - 49 years and being employed were risk factors associated with delayed diagnosis of tuberculosis [14]. In Ethiopia, diagnosis delayed risk factors were in 2005 [11], non-consultation in a formal health facility and self-medication; in 2012 [12], self-medication (aOR = 3.99), consultation of informal medicine (aOR = 6.18), and consultation in private structures (aOR = 19.70); and in 2020 [13], a distance of more than 10 km from the health center (OR = 3.23), self-medication or informal treatment (OR = 3.01), and the low level of TB know-ledge (OR = 1.58). In South Africa, risk factors for delayed diagnosis of TB among HIV-infected patients were age > 40 years 3.43-fold and virologic failure 2.72-fold [16]. Elsewhere in the world, in Nepal, smoking more than 5 cigarettes per day was a risk factor of a longer delay of more than 30 days [17]. In Cambodia, living in rural areas, hemoptysis and night sweats, self-medication, private center, and stigma were risk factors for delayed diagnosis [21]. In the USA, the Harvard Medical School study in private insurance holders found elderly and HIV-negative as associated risk factors of delayed diagnosis and conversely, the presence of ≥3 signs, consultation with a TB specialist was associated with a shorter delay, in addition, a longer delay was associated with lung complications [22]. These observations point out the need to increase TB knowledge in the population and train health workers to early request TB diagnostic tests and more specifically in private health practitioners.

#### Limitations:

The risk factors identified if considered can help to attain objectives toward END TB. However, the study had some limitations. The question on reasons that have contributed to the diagnosis delay was general and could have been more specific, thus clearly giving ways for the modification of the identified factors. In Mali, the time for widows to stay home after the death of their husbands goes from forty days to six months. Also, the study could have identified the qualification of the health personnel who first saw the participant. The national TB program recommends that any cough duration more than two weeks should be screened for TB.

## 5. Conclusion

This study has shown that the delay in diagnosis of tuberculosis is relatively very long, around four months in Mali. Several modifiable factors are associated with this delay, education below university, social reasons, and most importantly, the non-request of the diagnostic test by health workers. It can be drawn that patients are visiting late, and TB suggestive signs are not considered at the first consultation. To achieve the objective of 90% screening in Mali, a nationwide survey must be conducted by health workers to identify the reasons for medical and patient delays for TB diagnosis.

## Figures and Tables

**Table 1.** Sociodemographic and clinical characteristics of the sample.

Characteristics		Effective	Percentage
***Age range***	≤25 years	19	7.1
	26 - 50 years	200	75.2
	>50 years	47	17.7
***Sex***	Male	215	80.8
	Female	51	19.2
***Marital status***	Married	221	83.1
	Single/Widow	45	16.9
***Level of education***	Unschooled	38	14.30
	Primary	134	50.37
	Secondary	69	25.34
	University	25	09.40
***Current address***	Bamako	197	74.10
	Region of Mali	29	25.90
***Profession***	Official	24	09.02
	Trade	33	12.41
	Worker	101	37.97
	Farmer/Breeder	57	21.43
	Household	40	15.04
	Student	11	04.15
***monthly gain* (*CFA*)**	<100,000	75	28.20
	100,000 - 200,000	174	65.40
	> 200,000	17	06.4
***Smoking***	Yes	112	42.10
	No	154	57.90
***Degree of tobacco intoxication* (*n* = 112)**	0 - 10 pack/year	46	41.07
	11 - 20 pack/year	45	40.18
	21 - 30 pack/year	13	11.61
	>30 packs/year	8	07.14
***Clinical symptoms***	*Cough/Anorexia*	266	100
	*Dyspnea*	169	63.50
	*Chest pain*	203	76.30
	*Hemoptysis*	34	12.80
***Body Mass Index* (*BMI*) *kg*/*m*^2^**	<18.50	212	79.70
	18.5 - 24.9	53	19.9
	25.0 - 29.0	01	0.40
***WHO Performance Status* (*PS*)**	Score 1 or 2	176	66.17
	Score 3 or 4	90	33.83
***Chest X-ray findings***	Unilateral	132	49.60
	Bilateral	134	50.40
	Excavation	47	17.70

**Table 2.** Patient itinerary before TB diagnosis confirmation.

Itinerary	Effective	Percentage
*Self-medication and consultation with the healer*		
Yes	118	44.40
No	148	55.60
*Number of structures attended*		
one to two	141	53.0
Three or more	125	47.0
*Type of structure frequented*		
Community health center	162	60.90
Reference health center	104	30.10
Private health structure	106	39.85
Pharmacy	117	44.0
Hospital	11	04.14
*Test used for diagnosis*		
Microscopy	205	77.07
GeneXpert^®^ MTB/RIF	61	22.93
*Structures that requested the diagnosis*		
Referral health center	23	08.60
Hospital	243	91.40
*Reasons for delay in consultation or diagnosis*		
Financial	89	33.50
Personal	102	38.30
Social	82	30.83
Professional	81	30.50
Healthcare worker (medical)	112	79.70
*Cost of expenses before diagnosis*		
Less than 50,000 FCFA	44	16.5
50,000 - 100,000 FCFA	95	35.7
More than 100,000 FCFA	127	47.7
*Patient delay* (*in days*)		
0 - 30	56	21.05
31 - 60	163	61.28
≥61	47	17.67
*Medical delay* (*in days*)		
0 - 30	55	20.68
31 - 60	167	62.78
61 and over	44	16.54
*Diagnosis delay* (*in days*)		
1 - 90	77	28.95
120 - 150	185	69.55
≥150	4	01.50

**Table 3.** Comparison of cost spent before tuberculosis diagnosis between smokers and non-smokers.

Smoking	Cost spent (CFA Franc)			Total
	<50,000	50,000 - 100,000	Over 100,000	
No	26	66	62	154
Yes	18	29	65	112
Total	44	95	127	266

There is a statistically significant association between smoking and non-smoking patients on the cost spent before the diagnosis of tuberculosis (Chi 2 test = 9.54; p = 0.009).

**Table 4.** Factors associated with tuberculosis diagnosis delays after 100 days.

Risk factors	Items	Time to diagnosis < 100 days	Time to diagnosis ≥ 100 days	Odds Ratio (95% CI), Fisher Exact p-value
Sex	Male	64 (24.8)	151 (56.8)	1.04 (0.92 - 1.18), p = 0.60
	Feminine	13 (04,9)	38 (14.3)	
Age	≤40 years	45 (16.9)	32 (12.0)	1.28 (0.75 - 2.18), p = 0.41
	>40 years	99 (37.2)	90 (33.8)	
Smoking	Yes	34 (12.8)	78 (29.3)	0.89 (0.52 - 1.52). P = 0.68
	No	43 (16.2)	111 (41.7)	
Primary healthcare/Pharmacy	Yes	120 (45.1)	69 (25.9)	0.35 (0.20 - 0.60), p < 0.0001
	No	29 (10.9)	48 (18.0)	
Traditional therapy/Self-medication	Yes	73 (27.4)	116 (43.6)	2.24 (1.30 - 3.83), p = 0.004
	No	45 (16.9)	31 (11.7)	
Hemoptysis	Yes	1 (0,4)	33 (12.4)	16.07 (2.16 - 119.78), p < 0.0001
	No	76 (28.6)	156 (58.6)	
Dyspnea	Yes	25 (09.4)	144 (54.1)	6.66 (3.72 - 11.92), p < 0.00001
	No	52 (19.5)	45 (16.9)	
Personal reason	Yes	36 (13.5)	66 (24.8)	0.61 (0.36 - 1.05), p = 0.095
	No	41 (15.4)	123 (46.2)	
professional reason	Yes	54 (20.3)	58 (21.8)	1.04 (0.58 - 1.85), p = 1.00
	No	131 (49.2)	23 (08.6)	
Financial reason	Yes	117 (44.0)	60 (22.6)	2.17 (1.18 - 4.01), p = 0.015
	No	72 (27.1)	17 (06.4)	
Social reason	Yes	120 (45.1)	64 (24.1)	2.83 (1.46 - 5.51), p = 0.002
	No	69 (25.9)	13 (04.9)	
Health worker did not request TB test	Yes	31 (11.7)	64 (24.1)	2.17 (1.17 - 4.04), p = 0.018
	No	158 (59.4)	23 (08.6)	

**Table 5.** Independent risk factors of tuberculosis diagnosis delay beyond 100 days.

Risk factors	Items	Time to diagnosis < 100 days	Time to diagnosis ≥ 100 days	Adjusted Odds Ratio (aOR)	P-value
male sex	Male	64 (24.8)	151 (56.8)	0.8 (0.3 - 2.2)	0.621
	Female	13 (04.9)	38 (14.3)		
marital status	Married	31 (11.6)	14 (05.3)	1.161 (0.5 - 3.2)	0.774
	Not Married	158 (59.4)	63 (23.7)		
Education below university	University	6 (02.3)	19 (07.1)	9.7 (1.9 - 50.2)	0.007
	Less than University	183 (68.8)	58 (21.8)		
Financial constraints	Yes	117 (44.0)	60 (22.6)	1.3 (0.4 - 3.6)	0.672
	No	72 (27.1)	17 (06.4)		
Work constraints	Yes	131 (49.2)	54 (20.3)	2.1 (0.8 - 5.8)	0.154
	No	58 (21.8)	23 (08.6)		
Personal reasons	Yes	36 (13.5)	66 (24.8)	1.7 (0.7 - 4.1)	0.312
	No	41 (15.4)	123 (46.2)		
Social Reasons	Yes	120 (45.1)	64 (24.1)	3.4 (1.2 - 9.4)	0.021
	No	69 (25.9)	13 (04.9)		
Health workers never requested TB test	Yes	31 (11.7)	64 (24.1)	8.1 (2.8 - 22.9)	<0.0001
	No	158 (59.4)	23 (08.6)		
Consultation primary care/pharmacy	Yes	120 (45.1)	69 (25.9)	1.1 (0.5 - 2.5)	0.883
	No	29 (10.9)	48 (18.0)		
Smoking	Yes	34 (12.8)	78 (29.3)	1.8 (0.8 - 4.2)	0.171
	No	43 (16.2)	111 (41.7)		
Hemoptysis	Yes	1 (0,4)	33 (12.4)	4.4 (0.5 - 38.6)	0.185
	No	76 (28.6)	156 (58.6)		
Self-medication/traditional therapy	Yes	73 (27.4)	116 (43.6)	0.5 (0.2 - 1.0)	0.062
	No	45 (16.9)	31 (11.7)		
Bilateral radiological findings	Yes	82 (30.8)	50 (18.8)	1.6 (0.8 - 3.5)	0.206
	No	107 (40.2)	27 (10.2)		
Age > 40 years	≤40 ans	45 (16.9)	32 (12.0)	0.9 (0.5 - 1.9)	0.660
	>40 ans	99 (37.2)	90 (33.8)		
Cost of expenses more than 100 thousand CFA	Yes	105 (39.5)	34 (12.8)	0.5 (0.2 - 1.3)	0.155
	No	84 (31.6)	43 (16.2)		

Education level below university, social reasons, and non-request of TB test by the health personnel were identified as independent risk factors for a delayed diagnosis of more than 100 days.
